# Screening the Single Nucleotide Polymorphisms in Patients with Internal Carotid Artery Stenosis by Oligonucleotide-Based Custom DNA Array

**Published:** 2009-11-24

**Authors:** Kenji Nakai, Mayu Oyanagi, Jiro Hitomi, Kuniaki Ogasawara, Takashi Inoue, Masakazu Kobayashi, Keiko Nakai, Akira suwabe, Wataru Habano, Toshiaki Baba, Hiroshi Yoshida, Akira Ogawa

**Affiliations:** 1 Department of Laboratory Medicine; 2 Neurosurgery; 3 Anatomy and DNA Laboratories; 4 Iwate Medical University, Morioka, Japan 020-8505, R&D center, Nipro Co., Ltd; 5 Kusatsu, Shiga, Japan 525-0055

**Keywords:** atherosclerosis, carotid artery stenosis, polymorphism, plasminogen activator inhibitor 1; 5, 10-methylenetetra hydrofolate reductase

## Abstract

Early screening of individuals considered to be at risk for severe internal carotid artery (ICA) stenosis is an important strategy for preventing ischemic cerebral stroke. The purpose of this study is to screening candidate single nucleotide polymorphisms (SNPs) associated with severe ICA stenosis using a newly developed oligonucleotide-based custom DNA array. The subjects consisted of 47 controls and 46 patients with severe ICA stenosis (≥70%) who underwent carotid endarterectomy (CEA). Subjects gave informed consent and we obtained samples of blood and genomic DNA. We studied 8 candidate genes: renin-angiotensin system [angiotensinogen (AGT), angiotensin II receptor type 1 (AGTR1), nitric oxide synthase 3 (NOS3)]; growth factor [hepatocyte growth factor (HGF)]; transgelin (SM22); cytokine [chemokine receptor 2 (CCR2)]; coagulation-fibrinolysis system [5,10-methylenetetrahydrofolate reductase (MTHFR)]; and plasminogen activator inhibitor 1 (PAI-1). Genotyping of candidate SNPs was done with a line probe assay (LiPA) based on an oligonucleotide-based DNA array. Results: The allele frequency of PAI-1 –1965 delG (odds ratio (OR), 0.3; 95% confidence interval (CI), 0.2–0.6) and MTHFR (OR 1.3, 95% CI, 1.0–1.5) were significantly different between controls and cases with ICA stenosis by Fisher’s exact test. Multiple logistic analysis revealed that diabetes mellitus (DM), SNPs in PAI-1 –1965 delG and MTHFR were an independent risk for ICA stenosis. In conclusion, genetic factors of coagulation-fibrinolysis as well as diabetes mellitus (DM) were relevant in ICA stenosis.

## Introduction

Early screening of individuals at risk for severe internal carotid artery (ICA) stenosis is an important strategy for preventing carotid-related stroke. Given the current knowledge of DNA sequences, single nucleotide polymorphisms (SNPs) are likely to represent valuable resources. The risk of ICA stenosis may be influenced partly by a coupled pattern of inheritance. However, the standardization of genotyping of SNPs has not been fully established.

Two different strategies for the post-sequencing phase of the Human Genome Project have been proposed (Collins and Guyer, 1997). One plan involves the systematic identification of all common variations in human genes. The second strategy involves the use of very dense maps of single nucleotide polymorphisms (SNPs). At this stage of the post-sequence era, SNPs are likely to represent valuable therapeutic targets for medical research, since they may be associated with susceptibility to disease or pharmacological responsiveness (Collins and Guyer, 1997; Kruglyak, 1997). Thus, SNPs may play a role in many common diseases, and may well underlie the need for individualized patient therapy (Lander, 1996). Hence, a systematic method for cataloging human SNPs was proposed (Livak et al. 1995), and several surveys of this type have been reported (Cargill et al. 1999; Halushka et al. 1999; Ohnishi et al. 2000). Highly multiplexed genotyping methods are needed to support a comprehensive analysis of SNP-related genes in ICA stenosis. The purpose of this study is screening SNPs markers that associated ICA stenosis by oligonucleotide-based custom DNA array.

## Subjects and Methods

We studied 47 control individuals without ICA stenosis and 46 consecutive patients with severe ICA stenosis (≥70%) who underwent carotid endarterectomy (CEA). While 27 patients evinced ipsilateral carotid territory symptoms, 19 patients exhibited asymptomatic ICA stenosis. The assessments of the risk factors as well as the personal history of vascular events were collected at enrolment. The risk factors assessed were: hyperlipidemia (total cholesterol >220 mg/dL, triglyceride >150 mg/dL, HDL-cholesterol <40 mg/dL), hypertension (systolic and/or diastolic pressure >160/90 mm/Hg on more measurements), diabetes mellitus (HbA1c >6%), and overweight (BMI > 26). Carotid arteries were studied by ultrasound sonography to determine the type of plaque, internal media thickness (IMT), and degree of stenosis of plaques.

## Ultrasound Assessment of the Carotid Arteries

The ultrasound assessment of the extra-cranial carotid arteries was performed using a color doppler ultrasound sonograph instrument (GE Yokogawa, LOGIQ 500, Japan) equipped with an 11-MHz multifocal linear-array transducer. The scanning protocol entails the longitudinal scanning of the near and far wall of both carotid arteries at the distal common carotid artery, carotid bifurcation, and proximal internal carotid artery. The common carotid artery was defined as the portion 10 mm below the dilatation of the bulb; the bifurcation was defined proximally by this landmark; the internal carotid artery was defined as the portion 10 mm above the tip of the flow divider. IMT was defined as a focal or linear area with lumen projections ≤1.5 mm. All images with lumen projections >1.1 mm were considered as plaques. Ultrasonic plaque structure was defined according to the classification proposed by Gray-Weale et al. (1988). Carotid stenosis was evaluated using an electronic caliper [% stenosis = (total vessel diameter − residual diameter)/total diameter × 100]. All measurements of B-scan images were done in real time.

## DNA Sample

We obtained blood samples and genomic DNA with informed consent as overseen by the Ethical Committee of Iwate Medical University. Genomic DNA was prepared from white blood cells using a DNA extraction kit (WAKO Inc., Tokyo, Japan).

## Selection of Candidate Genes and SNPs

We selected 8 SNPs from candidate genes that may be related to atherosclerosis, including genes involved in the renin-angiotensin system, cytokine, growth factors, and the coagulation-fibrinolysis system (Table 1).

We designed polymerase chain reaction (PCR) primers to amplify the regions surrounding SNPs using computer software. Gene nomenclature and symbols were based on The Genome Database (GDB; http://www.gdb.org/). SNPs were identified using the Online Mendelian Inheritance in Man database (OMIM; http://www.ncbi.nlm.nih.gov/Omim/), the Human Gene Mutation Database (HGMD; http://archive.uwcm.ac.uk/uwcm/mg/hgmd/search.html), and the IMS-JST Japanese SNPs Database (http://snp.ims.u-tokyo.ac.jp/index_ja.html). We selected SNPs that were reported frequently in the Japanese population in cases where several polymorphisms were encountered. We obtained Genomic DNA sequences around SNP sites from GenBank (http://www.ncbi.nlm.nih.gov/).

## Oligonucleotide-Based DNA Array

**Figure 1.**
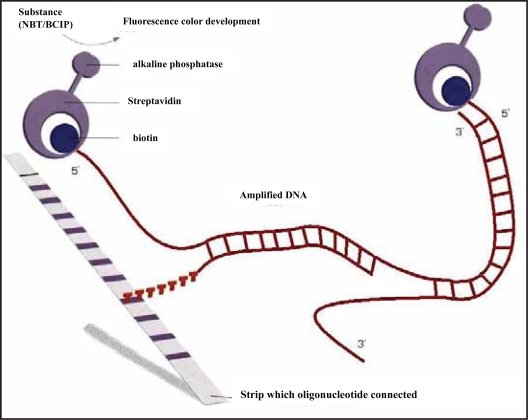
Schema for genotyping candidate SNPs (analyzed by LiPA) by reverse hybridization. Amplified biotinylated DNA material is hybridized with specific oligonucleotide probes immobilized as parallel lines on membrane-based strips. After hybridization, streptavidin labeled with alkaline phosphatase is added, and binds to any hybrid formed. After washing, incubation with nitro blue tetrazolium (NBT)/5-bromo-4-chloro-3-indolyl phosphate (*p*-toluidine salt) (BCIP) results in formation of a purple/brown precipitate.b.

The principle of the procedure is an oligonucleotide-based DNA array. Amplified biotinylated DNA material is hybridized with specific oligonucleotide probes immobilized as parallel lines on membrane-based strips. After hybridization, streptavidin labeled with alkaline phosphatase is added, and binds to any hybrid formed. After washing, incubation with BCIP/NBT chromogen results in a purple/brown precipitate (Fig. 1).

The details of the procedure are as follows. A volume (10 μL) of denaturation solution and 45 μL of amplified product were added into the upper corner of each well, mixed, and kept at room temperature for 5 minutes. Then the reaction mixture was added to 1 ml of warm (45 ºC) hybridization solution, and mixed gently. The strip was placed into a trough immediately, and incubated at 45 ºC on a shaker for 30 minutes (the following incubations were done on the shaker). After the reaction, 1 mL of warm stringent wash solution (SW) was added to each well, and it was rinsed for 10–20 seconds. A volume (1 mL) of SW was added to each well and incubated at 45 ºC for 10 minutes. Each strip was washed twice for 1 minute using 1 mL of rinse solution. A volume (1 mL) of conjugate solution was added to each well and incubated at room temperature for 30 minutes. Each strip was washed twice for 1 minute using 1 mL of rinse solution, and then washed using 1 mL of substrate buffer for 1 minute. Substrate solution (1 mL) was added to each well and kept at room temperature for 30 minutes. The color development was stopped by washing the strip in 1 mL of distilled water while agitating the tray on the shaker for 10 minutes. The strip was removed from the well and dried completely to detect genotypes from the coloring pattern.

## Genotyping of Candidate SNPs

**Figure 2.**
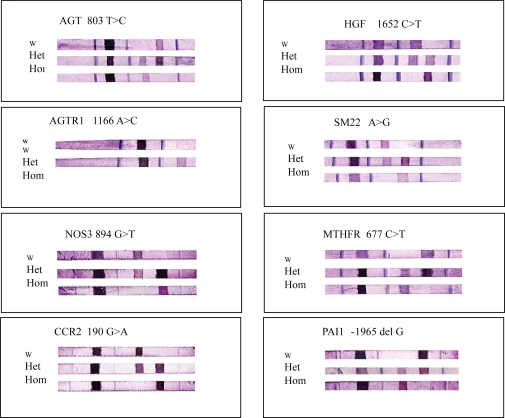
Representative genotyping of 8 SNPs analyzed using oligonucleotide-based DNA array (LiPA).

Genotyping of candidate SNPs was performed using an oligonucleotide-based DNA array (LiPA). Representative genotypes are presented in Figure 2. To evaluate the accuracy of the assay procedure, we determined the sequence of 8 SNPs in all patients by the fluorescent dye-terminator cycle method using an autosequencer (model 377XL, Applied Biosystems, Foster City, CA) and the primers that were used for PCR amplifications.

## Statistical Analysis

Data were also compiled according to the allele frequency and genotype. Statistical analyses involving odds ratios required calculation of the observed frequency of alleles and genotypes (dominant and recessive) in each group. Intergroup differences were tested by Fisher’s exact test. For all statistical analyses, *p* < 0.05 was considered significant. Multiple logistic regression tests, with the stepwise method, were carried out to determine the conditions influencing the progression of hypertension, dyslipidemia, diabetes, overweight, alleles and genotypes were considered as independent variables. We used SPSS (SPSS Inc., Tokyo, Japan) for multiple logistic analysis.

## Results

At the end of enrolment, 47 age-matched healthy controls and 46 consecutive patients were studied. Their general data and risk factors are given in Table 2.

The risk factors assessed (male, overweight, and hypertension) were not different between these two groups, except for hypertension and diabetes mellitus (DM), which had a lower prevalence in patients.

In 8 SNPs, there was complete agreement in sequence between the oligonucleotide-based DNA array (LiPA) and the fluorescent dye-terminator cycle method. A representative oligonucleotide-based DNA array is presented in Figure 2.

The allele frequency of PAI-1 –1965 delG (OR: 0.3, 95% CI, 0.2–0.6) and 5,10-methylenetetrahydrofolate reductase (MTHFR) (OR 1.3, 95% CI, 1.0–1.5) were significantly different between controls and cases with ICA stenosis by Fisher’s exact test. Stepwise multiple regression analysis was carried out to determine the association of known risk factors with ICA stenosis. The results are summarized in Table 3. Multiple logistic analysis revealed that DM, SNPs in PAI-1 –1965 delG and MTHFR were an independent risk for ICA stenosis, as shown in Table 4.

## Discussion

In the present study, we demonstrated that SNPs including coagulation-fibrinolysis analyzed oligonucleotide-based custom DNA Array as well as DM was relevant at risk in patients with ICA Stenosis.

Recent novel DNA sequencing approaches offer the prospect of identifying increasing numbers of SNPs at a reduced cost by high-throughput SNP discovery mature. Several promising methods for semi-automated or fully automated discovery or scoring of SNPs have been developed, including the Taqman approach, DNA chips, and the Invader assay (Kwiatkowski et al. 1999; Fan et al. 2000). DNA chips are very expensive for routine clinical use for screening SNPs in patients with ICA stenosis. In the present study, we used an oligonucleotide-based DNA array (LiPA). The oligonucleotide-based DNA array (LiPA) analyzed the 8 SNPs of interest with enough accuracy as confirmed by fluorescent dye-terminator cycle sequencing.

Previously, we reported 39 SNPs by chip-based MALDI-TOF MS using the MassARRAY system with a high level of accuracy at multiplex assays (Nakai et al. 2002). In 19 Japanese patients with myocardial infarction whose first attack occurred before the age of 50 years, significant differences were observed in the –1965 delG of PAI-1 with respect to allelic frequency, the G>A in the promoter region SNP in SM22 (TAGLN) for dominant genotype, and in two other SNPs (C>T in intron 1 of HGF, and –1965 delG of PAI-1) for recessive genotype. These surveys within candidate genes associated with coronary artery disease may prove relevant to studies of SNP-associated diseases in general.

In patients with 80–99% of stenosis in carotid artery, strokes occurred as well as contra-lateral to the plaque (Bock et al. 1993), and a high degree of stenosis was predictive of coronary events (Sillesen, 2002). Theoretically, the pathophysiological nature of the coronary artery and internal carotid artery may be similar. Therefore, we tested 8 SNPs to examine for a genetic risk factor for ICA stenosis. Among the 8 candidate genes, significant disequilibrium was observed in SNP-related genes of the PAI-1 –1965 delG allele, and MTHFR among controls and patients.

PAI-1, known to inhibit fibrinolysis in the circulation and to be present within atherosclerotic vessels, may influence proteolysis in the arterial wall. Sawa et al. (1994) demonstrated that gene and protein expression of plasminogen activator inhibitor type 1 (PAI-1) was increased from 1 to 4 weeks within the balloon injured carotid arteries. In addition, Kathiresan et al. (2005) demonstrated that two genetic variants, SNP rs2227631 and the PAI-1 –1965 delG (4G/5G polymorphism), were strongly associated (*p* < 0.0001) with PAI-1 levels among participants in the Framingham Heart Study. Thus, we speculated that the decreased cell-surface fibrinolytic activity likely to result from the increased PAI-1 expression may stimulate vascular smooth muscle cell proliferation. Furthermore, Bova et al. (2000) reported that alanine/valine (A/V) polymorphism at codon 677 of the MTHFR gene was associated significantly with severe CAS. We also demonstrated an increased concentration of homocysteine in alanine/valine polymorphism at codon 677 of the MTHFR gene and risk for myocardial infarction (Nakai et al. 2000).

Some of these gene polymorphisms, such as insertion/deletion (I/D) polymorphism of the ACE gene (Mattace-Raso et al. 2004), and apolipoprotein E (APOE) (Beilby et al. 2003) have been reported as a disease-candidate risk for ICA stenosis and coronary artery disease. Topic et al. (2001) showed an association of the MTHFR and APOE gene polymorphism with cerebrovascular disease, suggesting a significant risk for stroke in subjects who are homozygous for the T allele and for carotid stenosis in subjects having the APOE epsilon3/epsilon4 genotype.

Thus, genome-wide maps of SNPs might provide an efficient means of identifying new disease-susceptibility genes. Several surveys of this kind have been reported (Cargill et al. 1999; Halushka et al. 1999; Ohnishi et al. 2000). By cataloging dense maps of SNPs it may be possible to directly test the association of each SNP in individuals at high risk for ICA stenosis. In the present study, we demonstrated the the possibility of clinical utility of an oligonucleotide-based DNA array (LiPA).

## Study Limitation

Some important limitations should be recognized when interpreting the results of this study. First, the sample size was small, so that the potential effects of co-morbidities cannot be excluded. Second, more efficient and dense SNP arrays will be needed to screen for inheritance-based ICA stenosis in populations.

In conclusion, genetic factors of coagulation-fibrinolysis as well as diabetes mellitus (DM) were relevant in ICA stenosis. Oligonucleotide-based DNA arrays have led to the expectation that understanding the genetic basis of ICA stenosis.

## Figures and Tables

**Table 1.** Eight candidate SNPs and primer design.

**Gene**	**Gene symbol**	**Polymorphism**	**Primer 1**	**Primer 2**
*R-A System*				
Angiotensin I	AGT	803 >C	T: gctgctccc tgatggga	C: ctgacgggag ccagtgtgga
Angiotensin receptor 1	AGTR1	1166 A>C	C: aaatgagcc ttagctac	T: aaatgagca ttagctac
NOS3	NOS3	894 G>T	G: gatgag cccccag	T: cagatgatc ccccaga
*Cytokine*				
Chemokine receptor 2	CCR2	190 G>A	G: tggtcgt cctcatctt	A: atgctggt catcctca
*Growth factor*				
Hepatocyte growth factor	HGF	1652 C>T (intron1)	C: gaggac tgaaaaaagc	T: gaggattga aaaaagc
Transgelin	SM22-1	A>G (promoter region)	A: gctcacca agccacagg	G: gctcacca ggccacag
*Coagulation-fibrinolysis*				
5, 10-Methylenetetra hydrofolate	MTHFR	677 C>T	C: gcggg agtcgattt	T: cgggag ccgatttcat
Plasminogen activator inhibitor 1	PAI1	−1965 del G	4G: acacgtg gggagtca	5G: acacgtgg gggagtca

**Table 2.** Patient background.

	**CONTROL**	**ICA stenosis**
Number	47	46
AGE	63 ± 10	67 ± 8
BMI	23 ± 2.4	23 ± 3.5
SBP	136 ± 15	141 ± 17
DBP	79 ± 8	79 ± 11
TC	202 ± 25	190 ± 36
TG	135 ± 67	154 ± 114
HDL	51 ± 14	53 ± 19
HBA1C	5.2 ± 0.5	5.6 ± 0.8

**Table 3.** Summary of SNPs among healthy controls and patients with ICA stenosis.

**Gene symbol**	**Polymorphism**	**Control (***n* = **47)**			CEA *a(*n* = **46)**			*p*value*b					
		whomo	hetero	mhomo	whomo	hetero	mhomo	allele	RR	CI	recessive	RR	CI
*R-A system*													
AGT	803 >C	2	11	34	1	9	36	0.53		1			
AGTR1	1166 A>C	43	4	0	40	6	0	0.53		0.52			
NOS3	894 G>T	41	4	2	40	4	2	1		1			
*Cytokines and adhesion molecules*													
CCR2	190 G>A	19	27	1	24	19	1	0.63		0.301			
*Growth factor*													
HGF	1652 C>T	1	8	38	1	7	38	1		1			
SM22-1	A>G (promoter region)	17	15	15		14	17	15		0.76	0.66		
*Coagulation-fibrinolysis*													
MTHFR	677 C>T	30	10	7	16	23	7	0.04	1.25	1.02–1.53	0.001	1.8	1.17–2.88
PAI1	1965 del G	2	15	30	15	22	10	0.001	0.36	0.24–0.57	0.001	0.25	0.09–0.72

* a: Abbreviations: CEA: carotid artery endoartectemy; whomo: wild-type homologous; hetero: heterologous; mhomo: mutant homologous; allele: allele frequency; dominant: wild-type frequency; recessive: mutant frequency; RR: rerative risk; CI: 95% confidence in.

**Table 4.** Summary of multiple logistic analysis.

**Risk factor**	**beta**	*p***-value**	**RR**
DM	1.44	0.02	4.2
MTHFR hetero	1.45	0.01	4.4
PAI1 homo	1.67	0.01	0.2
